# Oxaliplatin plus leucovorin and 5-fluorouracil (FOLFOX-4) as a salvage chemotherapy in heavily-pretreated platinum-resistant ovarian cancer

**DOI:** 10.1186/s12885-018-5180-1

**Published:** 2018-12-19

**Authors:** Vincenza Conteduca, Giorgia Gurioli, Lorena Rossi, Emanuela Scarpi, Cristian Lolli, Giuseppe Schepisi, Alberto Farolfi, Delia De Lisi, Valentina Gallà, Salvatore Luca Burgio, Cecilia Menna, Andrea Amadori, Lorena Losi, Dino Amadori, Maria Paola Costi, Ugo De Giorgi

**Affiliations:** 1 Medical Oncology Department, Istituto Scientifico Romagnolo per lo Studio e la Cura dei Tumori (IRST) IRCCS, Via Piero Maroncelli 40, 47014 Meldola, FC Italy; 2 Biosciences Laboratory, Istituto Scientifico Romagnolo per lo Studio e la Cura dei Tumori (IRST) IRCCS, Meldola, Italy; 3 Unit of Biostatistics and Clinical Trials, Istituto Scientifico Romagnolo per lo Studio e la Cura dei Tumori (IRST) IRCCS, Meldola, Italy; 4 Medical Oncology Department, Campus Bio-Medico University, Via Alvaro del Portillo 200, 00128 Rome, Italy; 5 Division of Gynecologic Oncology, Department of Obstetrics and Gynecology, Morgagni-Pierantoni Hospital, Forlì, Italy; 6 Department of Life Sciences, University of Modena and Reggio Emilia, Via Campi 183, 41125 Modena, Italy

**Keywords:** FOLFOX-4, Fluorouracil, Topotecan, Ovarian cancer, Platinum resistance, Survival

## Abstract

**Background:**

The purpose of this study was to evaluate the clinical impact of oxaliplatin, leucovorin, and 5-fluorouracil (FOLFOX-4) chemotherapy in terms of the response rate, progression-free/overall survival (PFS/OS) and safety profile in patients with heavily pretreated recurrent epithelial ovarian cancer.

**Methods:**

Clinical data were reviewed in 29 patients who received FOLFOX-4 as more than third-line chemotherapy, consisting of 85 mg/m^2^ of oxaliplatin, 200 mg/m^2^ of leucovorin, and bolus 400 mg/m^2^ on day 1 of 5-fluorouracil, followed by a 22-h infusion of 600 mg/m^2^ of 5-fluorouracil for 2 consecutive days every 3 weeks. We also compared the efficacy and toxicity of FOLFOX-4 with that of topotecan, a standard treatment, given at a dosage of 1.5 mg/m^2^ every three weeks in 26 patients.

**Results:**

The median age of enrolled patients was 60 years (range 33 to 85). A median of 4 cycles (range 1–17) of FOLFOX-4 were administered. Complete response and partial response were observed in one (3.5%) and 5 (17.2.2%) patients, respectively, while stable disease was reported in 8 (27.6%) patients. Among all patients, grade 3–4 anemia, neutropenia, and thrombocytopenia were observed in 0 (0%), 5 (17.2%), and 3 (10.3%) cases, respectively. Grade 3–4 fatigue was recorded in one (3.4%) patient and diarrhea in 2 (6.9%). Median PFS and OS were 2.8 months [95% confidence interval (CI) 1.7–4.9] and 6.2 months (95% CI 2.4–14.6), respectively. No significant differences in terms of efficacy and toxicity were observed between patients receiving FOLFOX-4 and those treated with topotecan.

**Conclusions:**

The FOLFOX-4 regimen would seem to obtain similar survival rates to those of standard therapy with topotecan in platinum-resistant ovarian cancer. Further randomized trials are warranted to confirm our findings.

## Background

Ovarian cancer remains a highly lethal malignancy, representing the sixth leading cause of cancer death in women and the most lethal gynecologic malignancy [1, 2]. The prognosis for advanced ovarian cancer has improved over the last 10 years, especially thanks mainly to the introduction of more personalized therapeutic strategies. However, despite the high response rate to the standard carboplatin-paclitaxel first-line combination, most patients develop recurrent disease, with a median survival ranging from 12 to 24 months. Patients who progress on cisplatin or carboplatin or who relapse within 6 months of the end of treatment show the poorest outcome [2]. Single-agent therapies for platinum-refractory/resistant patients include oral etoposide, taxanes, topotecan, gemcitabine, vinorelbine, liposomal doxorubicin, and oxaliplatin, with response rates of around 15–20% and an overall survival (OS) of less than 12 months [3]. Research is now aiming to improve chemotherapy, identify novel, effective and well-tolerated agents, and overcome platinum resistance.

Cisplatin and carboplatin are the most common platinum compounds used to treat ovarian cancer. Oxaliplatin, a diaminocyclohexane platinum compound, has a different spectrum of activity and toxicity to that of other platinum agents and does not usually show cross-resistance with cisplatin and carboplatin in ovarian cancer [4, 5]. In previous phase II studies, oxaliplatin at a dose of 130 mg/m^2^ every 3 weeks was administered to patients with cisplatin- or carboplatin-refractory/resistant and taxane-pretreated ovarian cancer, obtaining response rates of 4.3–29.0% and a median OS of 9.5–15 months [6–9]. The combination of 5-fluorouracil with leucovorin given as intravenous bolus or continuous infusion has also been used to treat platinum-resistant/recurrent ovarian cancer but has shown limited clinical activity [10–14].

Some in vitro studies indicate a potential synergy between oxaliplatin and 5-fluorouracil/leucovorin [15]. The combination of these drugs (called FOLFOX) represents a standard chemotherapeutic regimen in the management of some advanced tumours [16–18]. Preliminary data on the use of this treatment in recurrent ovarian cancer have not been entirely satisfactory/have not been encouraging [19–22]. In the present study we evaluated the efficacy, in terms of clinical outcome, and toxicity of FOLFOX-4 in platinum-resistant ovarian cancer. In addition, through an exploratory analysis, we compared our results with data from patients treated with topotecan, a drug usually administered alone as salvage chemotherapy in platinum-resistant disease.

## Methods

### Study population

We retrospectively evaluated 29 patients treated with the FOLFOX-4 regimen from February 2008 to April 2016 as the primary cohort, and 26 patients treated with topotecan between August 2010 and December 2014 as the secondary cohort. Eligibility criteria of both cohorts were histological confirmation of epithelial ovarian cancer, previous treatment with cisplatin or carboplatin plus paclitaxel regimens, and disease recurrence during treatment with or within 6 months of the end of the cisplatin or carboplatin-based chemotherapy. Additional eligibility criteria included Eastern Cooperative Oncology Group (ECOG) performance status 0–2, and adequate cardiac, renal, hepatic and bone marrow function. Metastatic disease was documented by bone scan, computed tomography or magnetic resonance imaging. The retrospective study was approved by the Institutional Review Board of Istituto Scientifico Romagnolo per lo Studio e la Cura dei Tumori (IRST) IRCCS and was conducted in accordance with the ethical standards laid down in the 1964 Declaration of Helsinki. The need for written informed consent from participants was waived because of the retrospective nature of the research.

Patients were evaluated for safety and dosing compliance every 2 weeks for the first 3 months of chemotherapy, and then monthly thereafter until treatment discontinuation. Renal, liver and bone marrow function were assessed at every cycle, while cancer antigen 125 (CA-125) and radiographic evaluation were left to the discretion of the treating physician, but were usually performed after at least three months’ treatment.

### Treatment and evaluation

Treatment with FOLFOX-4 consisted of 85 mg/m^2^ of oxaliplatin as a 2-h infusion on day 1, 200 mg/m^2^ of leucovorin as a 2-h infusion on day 1, and bolus 400 mg/m^2^ of 5-fluorouracil on day 1, followed by a 22-h infusion of 600 mg/m^2^ of 5-fluorouracil for two consecutive days every three weeks. Topotecan was administered at a dosage of 1.5 mg/m^2^ by intravenous infusion daily for 5 consecutive days, starting on day 1 of a 21-day course. Both therapeutic regimens were administered continuously until there was evidence of either progressive disease (PD) or unacceptable toxicity. Prophylactic granulocyte-colony stimulating factor was only permitted for patients who developed grade 3–4 neutropenia or febrile neutropenia.

Tumor response was evaluated every three cycles by repeating baseline assessments using imaging studies (computed tomography and/or magnetic resonance imaging) according to the Response Evaluation Criteria in Solid Tumors (RECIST) for patients with measurable disease [23]. CA-125 was evaluated in recurrent disease using CA-125 response criteria developed by the Gynecologic Cancer InterGroup [24]. Toxicity was graded using the National Cancer Institute Common Terminology Criteria for Adverse Events (CTCAE), version 4 [25].

### Statistical analysis

All data were analyzed by descriptive statistics. Relationships between patient characteristics were testing using the Chi-square test for categorical variables and the median test for continuous variables. The Kaplan-Meier method was used to estimate PFS and OS, with two-sided 95% confidence intervals (95%CI). PFS was defined as the time from the start of FOLFOX-4 or topotecan until disease progression or last tumor evaluation or death from any cause. OS was defined as the time from the start of FOLFOX-4 or topotecan until death from any cause or last follow-up. Survival curves were compared using the log-rank test. Due to exploratory intent, no multiple testing correction was performed. All statistical analyses were carried out with SAS statistical software, version 9.4 (SAS Institute, Cary, NC, USA). A two-sided *P*-value < 0.05 was deemed statistically significant for all the analyses.

## Results

### Patient characteristics

The median age was significantly different in the FOLFOX-4 and topotecan populations (60 years [range 33–85] and 66 years [range 51–80]), respectively (*P* = 0.032). FOLFOX-4-treated patients showed a higher incidence of abdominal and extra-abdominal metastases (18 [62.1%] vs. 6 [23.1%]) (*P* = 0.004). All patients had previously received a median of 4 (range 1–17) and 3 (range 1–8) cycles of FOLFOX-4 and topotecan, respectively (*P* = 0.038). Fourteen (48.3%) and 6 (23.1%) patients had received more than 4 treatments before FOLFOX-4 and topotecan, respectively (*P* = 0.055), whose 2 (range, 1–4) including a platinum-based treatment in both FOLFOX-4 and topotecan groups. Among FOLFOX-4 patients receiving a prior platinum-based treatment, we reported a recurrent and refractory disease in 19 (65.5%) and 10 (34.5%) patients, respectively, whilst we observed a recurrent and refractory cancer in 17 (64.4%) and 9 (35.6%), respectively, in the topotecan-treated patients. In the FOLFOX-4 group, a prior treatment with topotecan was reported in 4 (13.8%) patients, and only one (3.8%) patient received a prior therapy with FOLFOX-4 in the topotecan group. Among pre-treatment laboratory parameters, the incidence of neutrophil-to-lymphocyte ratio (NLR) ≥ 3 was significantly higher in patients treated with FOLFOX-4 than in those receiving topotecan (15 [53.6%] vs. 7 [28.0%]) (*P* = 0.013). Patient characteristics are summarized in Table 1.

**Table 1:** Patient Characteristics

	FOLFOX-4 (*n* = 29)	Topotecan (*n* = 26)	*P*
	N (%)	N (%)	
Median age, years (range)	60 (33–85)	66 (51–80)	0.032
Histology			
Serous	22 (75.9)	22 (84.6)	
Non-serous	7 (24.1)	4 (15.4)	0.422
FIGO stage at presentation			
I-II	3 (14.3)	2 (12.5)	
III	16 (76.2)	11 (68.7)	
IV	2 (9.5)	3 (18.8)	0.529
Unknown/missing	8	10	
Grade			
I	5 (20.8)	7 (29.2)	
II	5 (20.8)	4 (16.7)	
III	14 (58.4)	13 (54.1)	0.612
Unknown/missing	5	2	
ECOG Perfomance status			
0–1	27 (93.1)	25 (96.1)	
2	2 (6.9)	1 (3.9)	0.622
Sites of metastasis			
Only abdominal	11 (37.9)	20 (76.9)	
Abdominal + extra-abdominal	18 (62.1)	6 (23.1)	0.004
Number of involved sites			
1	4 (13.8)	5 (19.2)	
2	10 (34.5)	10 (38.5)	
≥ 3	15 (51.7)	11 (42.3)	0.459
Median interval from initial diagnosis, months (range)	47 (11.5–248)	40.4 (9.7–1301)	0.129
Lines of previous treatments			
≤ 4	15 (51.7)	20 (76.9)	
> 4	14 (48.3)	6 (23.1)	0.055
Number of treatment cycles			
Median value (range)	4 (1–17)	3 (1–8)	0.038
Baseline NLR			
< 3	13 (46.4)	20 (80.0)	
≥ 3	15 (53.6)	5 (20.0)	0.013
Unknown/missing	1	1	
Baseline PLR			
< 210	13 (46.4)	18 (72.0)	
≥ 210	15 (53.6)	7 (28.0)	0.062
Unknown/missing	1	1	
Median baseline Hb, g/dL (range)	11.1 (8.3–15.0)	12.1 (8.9–14.3)	0.058
Median baseline Ca125, ng/mL (range)	289.9 (13.3–11,344.0)	100.2 (12.6–10,805.0)	0.259
Median baseline BMI, kg/m^2^ (range)	23.88 (15.24–32.04)	23.16 (19.53–30.30)	0.345

*Abbreviation. BMI* body mass index, *ECOG* Eastern Cooperative Oncology Group, *FIGO* International Federation of Gynecology and Obstetrics, *FOLFOX-4* oxaliplatin, leucovorin, and 5-fluorouracil, *Hb* hemoglobin, *NL* ,neutrophil-to-lymphocyte ratio, *PLR* platelet-to-lymphocyte ratio

### Treatment outcomes

**Fig. 1:**
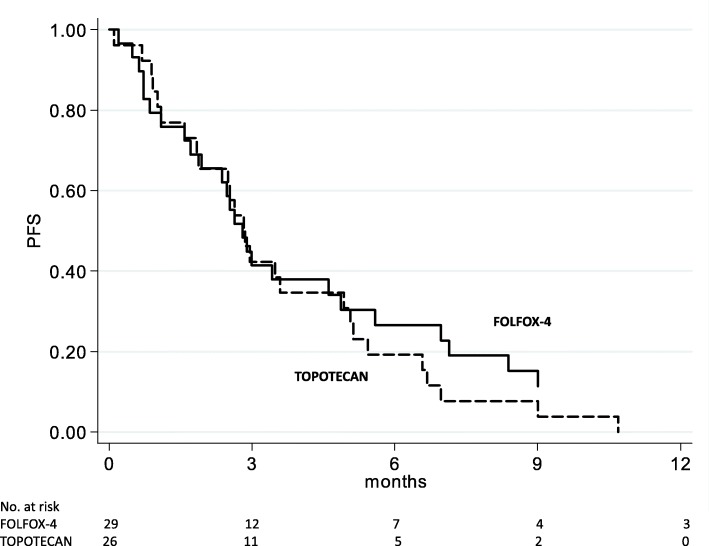
Progression-free survival (PFS) from start of FOLFOX-4 and topotecan treatments

**Fig. 2:**
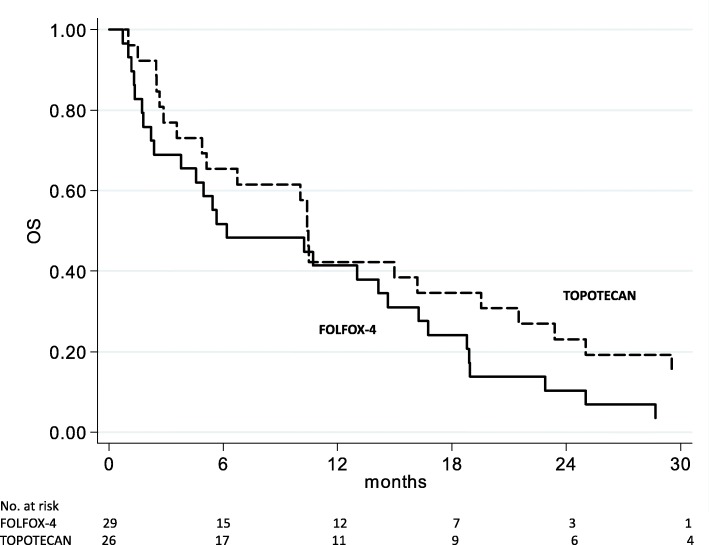
Overall survival (OS) from start of FOLFOX-4 and topotecan treatments

All patients treated with FOLFOX-4 and topotecan chemotherapy had measurable disease and were evaluable for tumor response by RECIST criteria (Table 2). Of the 29 patients treated with FOLFOX-4, 1 (3.5%) showed a complete response (CR), 5 (17.2%) a partial response (PR) and 8 (27.6%) stable disease (SD). Of the 26 topotecan patients, 1 (3.8%) had a CR, 1 (3.8%) a PR and 6 (23.1%) SD. Objective response was assessed by Rustin et al.’s [25] CA-125 criteria using the baseline CA-125 value as reference. Five patients treated with FOLFOX-4 and 8 with topotecan were not evaluable by Rustin’s CA-125 criteria. A CA-125 response was observed in 11 (44.0%) FOLFOX-4 patients and 5 (26.3%) topotecan patients (Table 2). The decrease in CA-125 levels was consistent with and not influenced by ascitic drainage. However, an objective radiological response (CR or PR) was not associated with CA-125 response. Median follow-up was 45 months (range 1–45) for patients treated with FOLFOX-4 and 57 months (range 1–57) for those receiving topotecan. The FOLFOX-4 group showed a median PFS and OS of 2.8 months (range 1.7–4.9) and 6.2 months (range 2.4–14.6), respectively. Topotecan patients had a median PFS and OS of 2.8 months (range 1.8–4.9) and 10.4 months (range 4.9–19.5), respectively (Table 2). A comparison of survival curves between the 2 treatment groups did not reveal a significant difference in PFS (Fig. 1) and OS (Fig. 2).

**Table 2:** Treatment outcome

	FOLFOX-4 (*n* = 29)	Topotecan (*n* = 26)
Median follow-up, months (range)	45 (1–45)	57 (1–57)
Median PFS, months (95% CI)	2.8 (1.7–4.9)	2.8 (1.8–4.9)
Median OS, months (95% CI)	6.2 (2.4–14.6)	10.4 (4.9–19.5)
Tumor response, *n* (%)		
CR	1 (3.5)	1 (3.8)
PR	5 (17.2)	1 (3.8)
SD	8 (27.6)	6 (23.1)
PD	15 (51.7)	18 (69.3)
Ca125 response*, *n* (%)	11 (44.0)	5 (26.3)
Number of patients receiving new treatment after progression, *n* (%)	16 (57.1)	17 (65.4)

*According to Rustin’s criteria

*Abbreviations. CR* complete response, *FOLFOX-4* oxaliplatin, leucovorin, and 5-fluorouracil, *n* number, *PFS* progression-free survival, *PD* progressive disease, *PR* partial response, *OS* overall survival, *SD* stable disease

Univariate analysis did not identify any significant factors (including site of metastasis, previous treatment lines, baseline NLR and platelet-to-lymphocyte ratio [PLR]) that predicted PFS (Table 3) and OS (Table 4) within each treatment group and between patients treated with FOLFOX-4 and topotecan, with the exception of NLR, a significant predictor of OS in FOLFOX-4 (*P* = 0.013).

**Table 3:** Univariate subgroups analysis of progression-free survival

	FOLFOX-4			TOPOTECAN			
	No. patients	No. events	Median PFS (95% CI)	No. patients	No. events	Median PFS (95% CI)	*P*
Site of metastasis							
Only abdominal	11	10	2.8 (0.6–5.6)	20	20	2.7 (1.1–3.6)	0.617
Abdominal+extra-abdominal	18	18	2.8 (1.7–7.0)	6	6	5.0 (0.9–10.7)	0.941
*P*			0.544			0.211	
Lines of previous treatments							
≤ 4	15	15	2.6 (0.6–4.6)	20	20	3.2 (1.6–5.4)	0.948
> 4	14	13	2.8 (1.7–8.4)	6	6	2.2 (0.7–4.9)	0.124
*P*			0.401			0.054	
Baseline NLR							
< 3	13	12	3.0 (2.4–18.5)	20	20	2.9 (1.6–5.1)	0.142
≥ 3	15	15	1.9 (0.7–4.6)	5	5	2.5 (1.0–6.6)	0.690
*P*			0.075			0.388	
Baseline PLR							
< 210	13	12	2.9 (0.7–18.5)	18	18	2.9 (1.6–5.1)	0.235
≥ 210	15	15	2.5 (0.9–4.6)	7	7	2.5 (0.9–3.5)	0.539
*P*			0.177			0.154	

*Abbreviation. FOLFOX-4* oxaliplatin, leucovorin, and 5-fluorouracil, *CI* confidence interval, *NLR* neutrophil-to-lymphocyte ratio, *PFS* progression-free survival, *PLR* platelet-to-lymphocyte ratio

**Table 4:** Univariate subgroups analysis of overall survival

	FOLFOX-4			TOPOTECAN			
	No. patients	No. events	Median PFS (95% CI)	No. patients	No. events	Median PFS (95% CI)	*P*
Site of metastasis							
Only abdominal	11	11	5.0 (1.3–14.6)	20	18	10.4 (2.9–25.0)	0.166
Abdominal+extra-abdominal	18	17	8.2 (2.4–16.7)	6	6	12.7 (2.7–23.4)	0.919
*P*			0.771			0.492	
Lines of previous treatments							
≤ 4	15	15	10.2 (1.3–16.7)	20	18	10.5 (2.7–25.0)	0.057
> 4	14	13	5.5 (2.4–14.6)	6	6	10.4 (2.9–16.2)	0.789
*P*			0.753			0.206	
Baseline NLR							
< 3	13	12	14.6 (3.8–22.9)	20	18	10.4 (4.9–23.4)	0.870
≥ 3	15	15	5.4 (1.2–10.2)	5	5	2.9 (1.5–15.0)	0.858
*P*			*0.013*			*0.051*	
Baseline PLR							
< 210	13	12	13.0 (1.8–18.9)	18	16	10.5 (4.9–23.3)	0.514
≥ 210	15	15	5.6 (1.3–14.1)	7	7	10.0 (1.5–15.0)	0.660
*P*			0.275			0.061	

*Abbreviations. FOLFOX-4* oxaliplatin, leucovorin, and 5-fluorouracil; number; *CI* confidence interval, *NLR* neutrophils-to-lymphocyte ratio, *OS* overall survival, *PLR* platelet-to-lymphocyte ratio

At the time of analysis, 1 (3.4%) patient in the FOLFOX-4 group and 2 (7.7%) in the topotecan group were still alive. After progression on FOLFOX-4 or topotecan, 16 (57.1%) and 17 (65.4%) patients, respectively, underwent new treatments (Table 2).

### Safety and tolerability

The incidence of grade 3–4 toxicity, in particular myelotoxicity, was similar in patients treated with FOLFOX-4 and topotecan (Table 5). Ten (34.5%) and 2 (7.7%) patients reduced FOLFOX-4 and topotecan dosage due to chemotherapy-related adverse events, respectively. However, only 2 (6.9%) undergoing FOLFOX-4 and 3 (11.5%) receiving topotecan discontinued treatment because of unacceptable toxicity.

**Table 5:** Toxicity in FOLFOX-4 and Topotecan cohorts

	FOLFOX-4 (*n* = 29)		Topotecan (*n* = 26)	
	Grade 3 *n*(%)	Grade 4 *n*(%)	Grade 3 *n* (%)	Grade 4 *n*(%)
Anemia	–	–	2 (7.7)	1 (3.8)
Neutropenia	3 (10.3)	2 (6.9)	–	–
Thrombocytopenia	1 (3.4)	2 (6.9)	2 (7.7)	1 (3.8)
Fatigue	1 (3.4)	–	1 (3.8)	–
Neurotoxicity	–	1 (3.4)	–	–
Hepatotoxicity	–	–	1 (3.8)	–
Diarrhea	2 (6.9)	–	–	–

*Abbreviations. FOLFOX-4* oxaliplatin, leucovorin, and 5-fluorouracil, *N* number

## Discussion

Despite relatively high response rates to first-line platinum-based therapies for epithelial ovarian cancer, the majority of patients relapse and a number of treatment-related deaths have also been reported. New-generation chemotherapeutic drugs and biological agents, especially those targeting angiogenesis [26] and the nuclear enzyme poly-(ADP-ribose) polymerase (PARP) [27, 28], have recently been introduced into clinical practice, increasing the number of therapies available for relapsed or refractory disease. Consequently, patients with advanced ovarian cancer often undergo multiple chemotherapy courses in an effort to achieve long-term remission and maintain an acceptable quality of life. The main risk from using an increasing number of therapeutic agents is cumulative toxicity, especially myelotoxicity, which may influence subsequent treatments. Hence the need for new, effective and less toxic therapies in patients with recurrent and persistent disease after failure of chemotherapy.

In the present retrospective monoinstitutional study, we analyzed the results obtained in a population of ovarian cancer patients treated with FOLFOX-4 or standard topotecan monotherapy in terms of clinical impact on outcome and toxicity. The choice of therapy and dosing schedule was at the discretion of the treating physician, as was the possibility of initial dose reduction due to older age and poor performance status. Efficacy and safety were comparable in both regimens, with hematological toxicity the most frequent reason for dose reduction. Treatment discontinuation due to toxicity was rare. Despite dose limiting cumulative neurotoxicity of oxaliplatin-based therapy, only one case presented a grade 4 neurotoxicity, although grade 2 neurotoxicities had a negative impact on the quality of life in a few cases heavily pretreated with taxane- and platinum-based therapies.

In terms of response, our findings were comparable to those of other studies [19–22] on patients with measurable disease. Major limitations of this study were a small number of patients, the retrospective design, and the presence of mismatch of the cohorts related to a lack of randomization. We observed that NLR was the only prognostic factor in our patient cohort, as reported in a recent meta-analysis [29]. However, due to exploratory intent, no multiple testing correction was performed. Although adjustments for multiple comparisons can help control the study-wide false-positive rate, for exploratory analyses it is more important to judge *P* values cautiously than to try to formally determine their true significance level. Precise adjustment of P values and confidence intervals is often impractical in the context of exploratory research but can be useful for hypothesis-driven research.

Despite all these several limitations of this study, we showed similar treatment outcomes (PFS, OS, and CA-125 response) between FOLFOX-4 and topotecan group. Specifically, there were fewer cases of PD (15 [51.7%] vs.18 [69.3%]) and a similar number of SD (8 [27.6%] vs. 6 [23.1%]) in FOLFOX-4 compared to topotecan patients. FOLFOX-4 could thus represent a potential alternative to standard chemotherapy, with a similar toxicity profile, in this patient setting. However, more recent studies [30] suggested different dose regimes of topotecan characterized by reduced number of side effects and thus this could alter the comparison of toxicities between FOLFOX-4 and topotecan profiles in our study.

The present study did not bring to light any clinical prognostic factors for PFS and OS in either treatment group, probably because of the small sample size and the lack of adequate patient selection. In addition, currently, there are no biomarkers able to improve the selection of patients candidates to FOLFOX-4 combination. Potential predictive biomarkers could derive from the analysis of homologous recombination deficiencies such as BRCA1/2 alterations, especially because they are of particular interest for platinum-based regimens. Therefore, future clinical trials in this disease setting could be supported by genomic and proteomic studies to identify prognostic factors associated with response to fluorouracil. The advances made in genetic and molecular biology could provide a valuable insight into the alterations underlying these types of ovarian cancer, and the relationship between the mechanism of action of fluorouracil and the subsequent downstream molecular pathways activated during tumorigenesis and disease progression.

## Conclusion

The retrospective nature of our study and the small sample size do not allow for definitive conclusions to be drawn. However, our results provide further evidence that the FOLFOX-4 regimen may be as effective as standard monotherapies and could be proposed as an appropriate salvage treatment in refractory or resistant ovarian cancer. However, it should be appropriate to consider these heavily-pretreated patients as an ideal group for clinical trials; particularly given the success or emerging data of some newer classes of targeted therapies such as PARP inhibitors, antivascular drugs, dual antibody like molecules, antibody drug conjugates and so randomized multicenter trials comparing the FOLFOX-4 regimen, alone or, particularly, in combination with targeted therapy, are warranted to improve the standard of care in patients with heavily-pretreated disease.

## Abbreviations

95%CI: 95% confidence interval
CA-125: cancer antigen 125
CR: complete response
CTCAE: Common Terminology Criteria for Adverse Events
ECOG: Eastern Cooperative Oncology Group
FOLFOX-4: oxaliplatin plus leucovorin and 5-fluorouracil
NLR: neutrophil-to-lymphocyte ratio
OS: overall survival
PARP: poly(ADP-ribose) polymerase
PD: progressive disease
PFS: progression-free survival
PLR: platelet-to-lymphocyte ratio
RECIST: Response Evaluation Criteria in Solid Tumors
SD: stable disease
